# A novel low-parameter computational model to aid *in-silico *glycoengineering

**DOI:** 10.1186/1753-6561-9-S9-P26

**Published:** 2015-12-14

**Authors:** Philipp N Spahn, Anders H Hansen, Henning G Hansen, Johnny Arnsdorf, Helene F Kildegaard, Nathan E Lewis

**Affiliations:** 1 Department of Bioengineering, University of California, San Diego, La Jolla, CA 92093, USA; 2 School of Medicine, Department of Pediatrics, University of California, San Diego, La Jolla, CA 92093, USA; 3 The Novo Nordisk Foundation Center for Biosustainability at the University of California, San Diego School of Medicine, San Diego, La Jolla, CA 92093, USA; 4 The Novo Nordisk Foundation Center for Biosustainability, Technical University of Denmark, Hørsholm, Denmark

## Background

**Figure 1:**
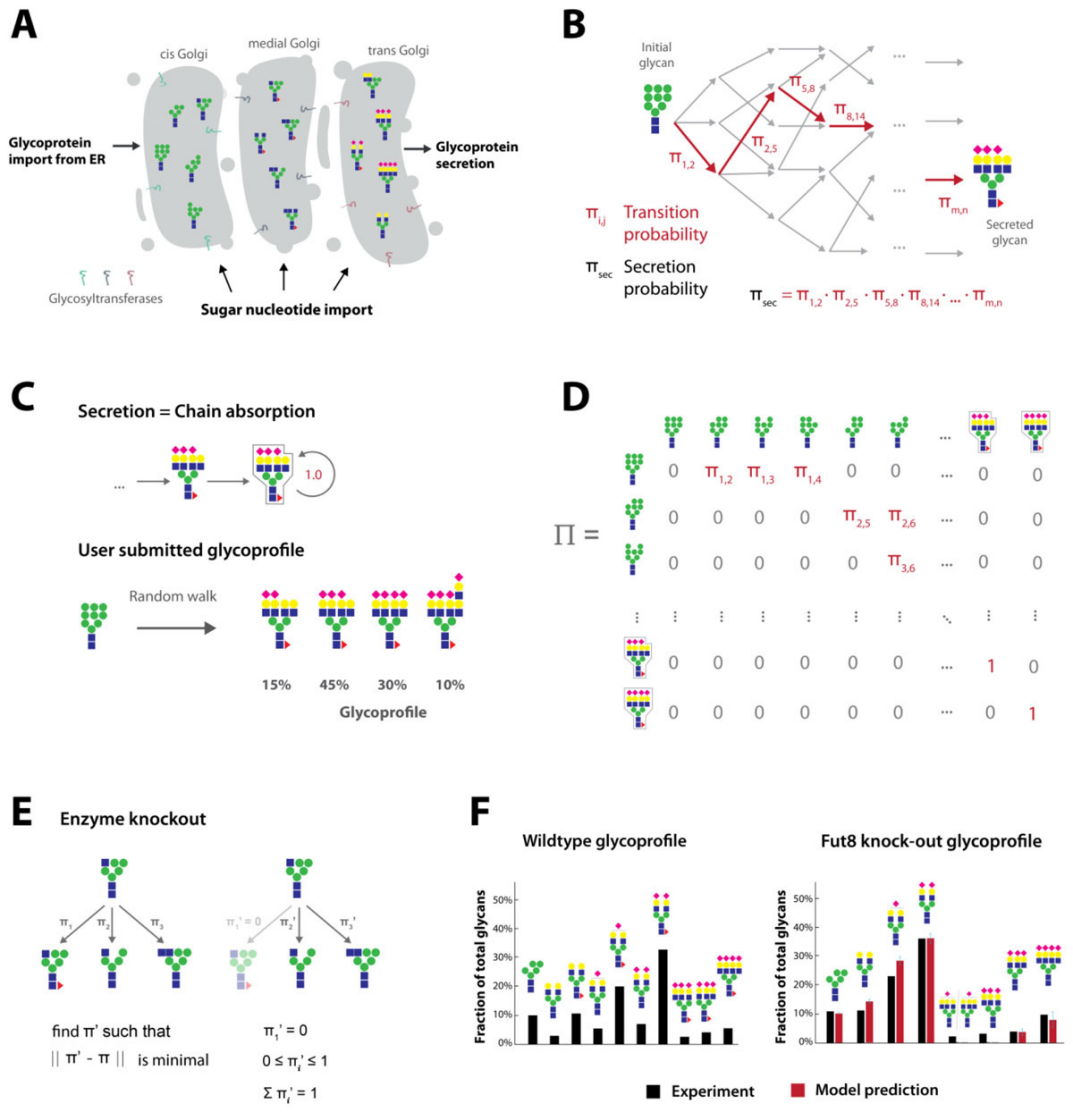
****(a)**Glycosylation is the outcome of a complex reaction network where the glycoprotein is successively processed by several enzymes in a sequence of Golgi compartments**.**(b)**In the model glycosylation is conceptualized as a random walk (Markov chain) starting in the initial Man_9_GlcNAc_2_ glycan and reaching a certain secreted glycan with a certain secretion probability. **(c) **Secreted glycans are modelled as absorbing states of the Markov chain that transition to themselves with probability 1, once they have been reached. To fit the unknown transition probabilities, the user inputs the experimentally derived glycoprofile of the protein of interest. **(d) **The deduced transition probabilities are arranged in a Markov transition matrix that gives the probabilities of transitioning from any glycan (rows) to any other (columns). (e)To model an enzyme knockout, the corresponding transition probability is set to 0. Optimization is used to adjust the alternative transition probabilities to maintain a probability sum of 1 for every glycan affected by the knockout. **(f)**Wildtype and Fut8 knockout glycoprofile of CHO-S secretome (black bars), together with the glycan frequencies as predicted by the model (red bars).

Glycosylation is a key post-translational modification that can affect critical properties of proteins produced in biopharmaceutical manufacturing, such as stability, therapeutic efficacy or immunogenicity. However, unlike a protein's amino acid sequence, glycosylation is hard to engineer since it does not follow any direct equivalent of a genetic code. Instead, its complex biogenesis in the Golgi apparatus (Figure 1A) integrates a variety of influencing factors most of which are only incompletely understood. Various attempts have been undertaken so far to computationally model the process of glycosylation, but due to the high parametric demand of most of these models, it has been challenging to leverage these models for glycoengineering purposes. Consequently, industrial glycoengineering is still largely carried out using costly and time-consuming trial-and-error strategies and could greatly benefit from computational models that would better meet the requirements for industrial utilization. Here, we introduce a novel approach combining constraints-based and stochastic techniques to derive a computational model that can predict the effects of gene knockouts on protein glycoprofiles while requiring only minimal a-priori parameter input.

### Computational approach

We use the COBRA toolbox to generate an in-silico representation of the N-glycosylation network. The stochastic transition of glycans through this reaction network is modeled as a Markov chain where secreted glycans are represented as absorbing states (Figure 1B,C). After the user has submitted an experimentally derived glycoprofile on a specific protein (for instance, obtained from a cell culture grown under standard conditions, Figure 1C), sampling methods are used to deduce the unknown probabilities of transitioning from one glycan to another in the network. These transition probabilities are concisely assembled in a Markov transition matrix (Figure. 1D). After this fitting procedure, enzyme knockouts are modelled by setting particular transition probabilities to zero and adjusting the remaining probabilities through optimization (Figure 1E).

## Results

Our model is capable of creating N-glycosylation reaction networks that are complex enough to cover typical glycoprofiles found in biopharmaceutical manufacturing including tetra-antennary, highly sialylated or polylactosamine carrying glycans. The probabilistic framework implemented in this model proves to outperform knockout predictions derived from pure constraints-based modeling. Tests on experimental knockout glycoprofiles both from the literature and our laboratory show that the model yields sound predictions of glycoprofile change upon genetic modification which are in good congruence with corresponding experiments (Figure 1F).

## Conclusion and outlook

The model has the potential to provide a cheap and fast guidance tool to help find host conditions that can yield a desired glycoprofile, thus providing an important step towards the in-silico process of glycoengineering. So far, the reaction network considered is specific for CHO cells but can be easily modified to include reactions occurring in other hosts. In addition, it could be integrated into whole-cell metabolic models. This would enable comprehensive in-silico representations of the entire cell-culture setup, allowing one to simulate the effects on the glycoprofile of a wide range of both intracellular and extracellular modifications to the growth conditions.

## Acknowledgements

This work was funded from a generous gift from the Novo Nordisk Foundation to the Center for Biosustainability. A provisional patent has been filed concerning this work. In addition, we wish to thank H. Clausen and his group from Copenhagen University for valuable discussions and sharing unpublished data.

